# Fluorescence in situ hybridization is superior for monitoring Epstein Barr viral load in infectious mononucleosis patients

**DOI:** 10.1186/s12879-017-2412-y

**Published:** 2017-05-03

**Authors:** Pengfei Cao, Meili Zhang, Wei Wang, Yafei Dai, Buqing Sai, Jun Sun, Lujuan Wang, Fan Wang, Guiyuan Li, Juanjuan Xiang

**Affiliations:** 1 The Key Laboratory of Carcinogenesis of the Chinese Ministry of Health, Xiangya Hospital, Central South University, Changsha, Hunan China; 2 The Key Laboratory of Carcinogenesis and Cancer Invasion of the Chinese Ministry of Education, Cancer Research Institute, Central South University, Changsha, Hunan China; 3 Department of hematology, Xiangya hospital, Central South University, Changsha, China; 4 Hunan Key Laboratory of Nonresolving Inflammation and Cancer, Disease Genome Research Center, The Third Xiangya Hospital, Central South University, Changsha, Hunan China; 5 People’s Hospital of Dezhou, Dezhou, Shandong 253045 China

**Keywords:** EB viral load, Infectious mononucleosis, Real-time PCR, FISH

## Abstract

**Background:**

Epstein Barr virus (EBV) plays a causal role in some diseases, including infectious mononucleosis, lymphoproliferative diseases and nasopharyngeal carcinoma. Detection of EBV infection has been shown to be a useful tool for diagnosing EBV-related diseases. In the present study, we compared the performance of molecular tests, including fluorescence in situ hybridization (FISH) and EBV real-time PCR, to those of serological assays for the detection of EBV infection.

**Methods:**

Thirty-eight patients with infectious mononucleosis (IM) were enrolled, of whom 31 were diagnosed with a mild type, and seven were diagnosed with IM with haemophagocytic lymphohistiocytosis and chronic active EBV infection. Twenty healthy controls were involved in the study. The atypical lymphocytes in peripheral blood were detected under a microscope and the percentage of positive cells was calculated. EBV DNA load in peripheral blood was detected using real-time PCR. The FISH assay was developed to detect the EBV genome from peripheral blood mononuclear cells (PBMC). Other diagnosis methods including the heterophil agglutination (HA) test and EBV-VCA-IgM test, to detect EBV were also compared. SPSS17.0 was used for statistical analysis.

**Results:**

In all, 5–41% atypical lymphocytes were found among the PBMC in mild IM patients, whereas 8–51% atypical lymphocytes were found in IM patients with haemophagocytic lymphohistiocytosis and chronic active EBV infection patients. There was no significant difference in the ratios of atypical lymphoma between patients of the different types. We observed that 71.2% of mild IM patients and 85.7% of IM patients with haemophagocytic lymphohistiocytosis and chronic active EBV infection patients were positive for EBV-VCA-IgM. EBV-VCA-IgM was negative in all healthy control subjects. In addition, 67.1% of mild IM patients tested heterophile antibody positive, whereas 71.4% of IM patients with haemophagocytic lymphohistiocytosis and chronic active EBV infection tested positive. EBV DNA detected using real-time PCR was observed in 89.5% of these IM patients. The EBV genome was detected by the FISH assay in 97.4% of the IM patients. The EB viral loads detected by FISH and real-time PCR increased with the severity of IM. The EBV genome was detected in almost all the PBMC of IM with haemophagocytic lymphohistiocytosis and chronic active EBV infection patients.

**Conclusion:**

Molecular tests, including FISH and EBV real-time PCR, are more sensitive than serological assays for the detection of EBV infection. The FISH assay detecting EBV copies in unfractionated whole blood is preferable and superior to plasma real-time PCR in its reflection of the absolute viral burden circulating in the patients.

## Background

Epstein Barr virus (EBV) is a γ-herpesvirus that infects at least 90% of the population worldwide [1]. In a majority of individuals, EBV infection is asymptomatic. EBV infection in adolescents and young adults frequently results in infectious mononucleosis (IM). IM presents as a mild EBV infectious illness and is self-limiting. However, EBV infection can also cause IM with haemophagocytic lymphohistiocytosis (HLH) or chronic active EBV infection (CAEBV). Both EBV-HLH and CAEBV are life-threatening complications [2]. Factors involved in the occurrence of IM with severe complications include immune status and polymorphisms in HLA-A01 or interleukin 10 [3, 4].

The clinical presentations, presence of atypical lymphocytes in peripheral blood, and positive heterophile antibody test are used for the diagnosis of IM. IM is not always considered early on; in particular, when there is a high level of viral upper-respiratory infections, diagnosis of mild EBV infection is difficult, if not impossible [5]. In patients with prolonged or vague symptoms, the diagnosis of acute IM can be easily missed. Rapid diagnosis can help the clinician become proficient with treatment. Serology tests are the main diagnostic strategy for the detection of EBV. Viral capsid antigens (VCAs), early antigens (EAs), and EBNAs encoded by the EBV genome are mostly used for serodiagnosis [6]. Serological tests for measuring antibodies to EBV are not usually positive until symptoms have been present for one week or more. False positive and false negative results are frequently observed [7]. High antibody titers against EBV-related proteins are not necessary for diagnosis because there can be a lack of serologic response in patients with a CAEBV infection [8]. Heterophile antibodies are not specific and do not develop in some patients. Only 59–81% of IM patients over age 13 were positive for the heterophile antibody, and levels were even lower in children under age 13, especially early in the course of the illness [9]. Other EBV-related illnesses can also occur, including cancers such as Burkitt’s lymphoma and autoimmune diseases. Severe and lasting disease can develop in the form of CAEBV or HLH in EBV-related malignancy, which shows markedly elevated levels of EBV DNA in the peripheral blood. However, the plasma levels of EBV DNA cannot simply be linked to the severity or spread of infection, because high levels of EBV DNA were found in the blood of both asymptomatic and exacerbated IM [10]. The link between the EB viral load and severity of symptoms has not yet been determined.

The measurement of EBV DNA in peripheral blood has been shown to be a useful tool for the diagnosis of EBV-related diseases. It has been suggested that quantitative viral load assessment is superior to qualitative detection [11]. Quantitative real-time EBV PCR performed using the plasma of patients resulted in an increase in the definitive diagnosis of primary EBV infection [12]. In situ hybridization detects EBV-encoded RNAs (EBERs) and is considered a good test for localizing latent EBV in tissue samples [7]. Here, we develop a new fluorescence in situ hybridization (FISH) probe with more than 3000 bp to detect cellular EBV DNA. This technique allows for the quantitative analysis of EBV copy numbers in infected cells. This method can evaluate the severity of EBV infection and may be used as a marker for treatment.

### Patients, materials and methods

1. Patients
  Patients newly diagnosed with IM (*n* = 38) were included in this study. Twenty healthy people were enrolled as a control group. All cases enrolled in this study were identified between January 2013 and December 2015 at Xiangya Hospital, Central South University, China. The diagnosis of IM was made by clinical presentation, heterophile antibody test, EBV serological determinations and serum EBV-DNA detected by real time-PCR. Patients diagnosed with HLH or CAEBV were identified by persistent infectious mononucleosis–like symptoms. Thirty-one patients with mild IM suffered from fever, hepatosplenomegaly, lymphoadenopathy and elevated transaminase levels. In addition to the common symptoms, jaundice, hepatic dysfunction, and severe cytopenia could be seen in the seven patients with HLH or CAEBV. The clinical characteristics of the 38 patients are listed in Table 1. The patients were informed about the sample collection and signed informed consent forms. Collection and use of samples were approved by the ethical review committees of Xiangya Hospital, Central South University. Table 1 the characteristics of IM patients
2. Atypical Lymphocytes
  Human peripheral blood mononuclear cells (PBMCs) were isolated from healthy donors and infectious mononucleosis patients using density gradient centrifugation. The atypical lymphocytes were detected using Wright staining (Baso Company, China). The atypical lymphocytes in the peripheral blood were detected under microscope, and the percentage of positive-stained cells was calculated.
3. Heterophil Agglutination test
  Sera from patients and healthy control were tested for heterophil antibodies using sheep erythrocyte agglutination. All sera were inactivated. The serum of each patient was absorbed with an equal volume of washed sheep erythrocytes. Any agglutination was considered to be due to IM heterophile antibodies.
4. Detection of peripheral blood EBV - VCA - IgM
  A recombinant immunofluorescent antibody (RIFA) test was performed to quantitatively detect human serum IgM antibodies to EBV-VCA (Focus Diagnostics, USA). The experiments were conducted in accordance with the manufacturer’s instructions. In brief, after incubation with appropriately diluted serum, the slides prepared with recombinant EBV VCA antigen were washed with phosphate buffered saline, and fluorescence in isothiocyanate-conjugated anti-human IgM was applied to each well at the appropriate concentrations. The slides were mounted with buffered glycerol and observed under a fluorescence microscope.
5. Real-time PCR for EBV
  Quantitative real-time EBV PCR was performed in plasma samples collected from IM patients and healthy controls using an Epstein-Barr virus DNA Quantitative Fluorescence Diagnostic Kit (Sansure Biotech, Hunan, China). Peripheral blood was obtained from patients when they presented at the hospital. Viral DNA was extracted, and the PCR reaction was performed according to the instructions. The qPCR protocol was 94 °C for 5 min, followed by 45 cycles of 94 °C for 15 s and 57 °C for 30 s. The EBV copy number was calculated according to the standard curve.
6. Cell Culture
  The human Burkitt lymphoma cell lines P3HR-1, RAJI and BJAB were cultured in RPMI-1640 (HyClone, Life Sciences, USA) supplemented with penicillin G (100 U/mL), streptomycin (100 mg/mL) and 10% foetal calf serum. Cells were grown at 37 °C in a humidified atmosphere of 5% CO_2_ and routinely sub-cultured using a 0.25% (*w*/*v*) trypsin-EDTA solution.
7. Fluorescence in situ hybridization
  An EBV-specific probe was prepared from EBV, which was obtained from the productive EBV B-cell lineage p3HR-1. The culture media were collected from P3HR-1. After repeated freezing and thawing, the collected media were centrifuged for 20 mins at 3000 RPM. The supernatant was collected and filtered with a 0.45-μm membrane filter. EB viral DNA was extracted from a sample aliquot of 250 μL using the Qiagen QIAamp Virus MinElute Spin Kit (Qiagen, Valencia, CA, USA) and then immediately frozen until use. DNA probes of 3267 bp long were generated via PCR.

**Table 1:** The characteristics of IM patients

	MILD	IM-HLH or IM-CAEBV	*P*-value
	(*n* = 31)	(*n* = 7)	
Gender			>o.o5
male	18(58.1%)	3(42.9%)	
female	13(41.9%)	4(57.1%)	
Age			>o.o5
0–14	24(77.4%)	5(71.4%)	
>14	7(22.6%)	2(28.6%)	
Leukocyte count			>o.o5
4 × 10^9^/L	7(22.6%)	2(28.6%)	
4–10 × 10^9^/L	8(25.8%)	1(14.3%)	
≥10 × 10^9^/L	16(51.6%)	4(57.1%)	
Median(range)	10.85(1.2–43.0)	14.9(1.5–61.0)	
ALT or AST			>o.o5
high	27(87.1%)	7(100%)	
normal/low	4(12.9%)	0(0%)	
coagulopathy			>o.o5
positive	22(74.2%)	6(85.7%)	
negative	9(25.8%)	1(14.3%)	
ferritin high			>o.o5
high	24(77.4%)	6(85.7%)	
normal/low	7(22.6%)	1(14.3%)	
Triglyceride			>0.05
high	24(77.4%)	6(85.7%)	
normal/low	7(22.6%)	1(14.3%)	
hepatosplenomegaly			>o.o5
yes	22(71.0%)	6(85.7%)	
no	9(29.0%)	1(14.3%)	
lymphoadenopathy			>o.o5
yes	24(77.4%)	5(71.4%)	
no	7(22.6%)	2(28.6%)	
Days after onset of disease			<o.o5
average time	34.8d	119.7d(4/7, 3 decreased)
  Forward primer: 5′-TTCGTCTTGCTCTATTCACCCTTAC-3′(EBV genome 5 ~ 28);
  Reverse primer: 5′-CACTGTAATGAAGACGTTGGAACAG-3′(EBV genome of 3271 ~ 3247).
  The PCR reaction was as follows: 94 °C for 1 min and 30 cycles of 94 °C for 30 s, 1 min 55 °C, 72 °C for 5 min, and 72 °C for 10 min. The 3267-bp PCR product was collected using a QIAquick PCR Purification Kit (Qiagen, Valencia, CA, USA) and labelled with biotin-dUTP using a randomly-primed labelling method (Roche, Mannheim, Germany). The reaction was terminated by heating at 65 °C for 10 min. PBMCs from patients were prepared and smeared on the slides. The slides were directly immersed in water (80 °C) for 5 min, denatured in 70% fomamide-2XSSC at 70 °C for 2 min and dehydrated in a series of ethanol concentrations, followed by air drying. The hybridizaiton mixture containing 50 ng of probe and 5μg of salmon sperm DNA was heated at 78 °C for 5 min to denature the probe, followed by preannealing at 37 °C for 1 h. The slides were incubated with the hybridization mixture overnight at 37 °C for 14 h. Following hybridization, the slides were washed in 50% formamide/2XSSC and then 0.1XSSC, followed by washing with 0.05% Triton X-100 /2 × SSC at 42 °C. The blocked slides were incubated with avidin-FITC for 30 min at room temperature, followed by incubation with anti-FITC. After washing, drying and mounting, the slides were examined under fluorescence microscopy. The cut-off value was based on the results of twenty healthy people in the control group. Two hundred cells in every control member and every patient were observed to calculate the average number of fluorescent particles in each cell. A fluorescent particle number less than the cut-off value (4–5 particles/cell) was considered negative.
8. Statistical analysis
  Statistical analyses were performed using SPSS 17.0. A *p* value of less than 0.05 was considered significant.

## Results

**Fig. 1:**
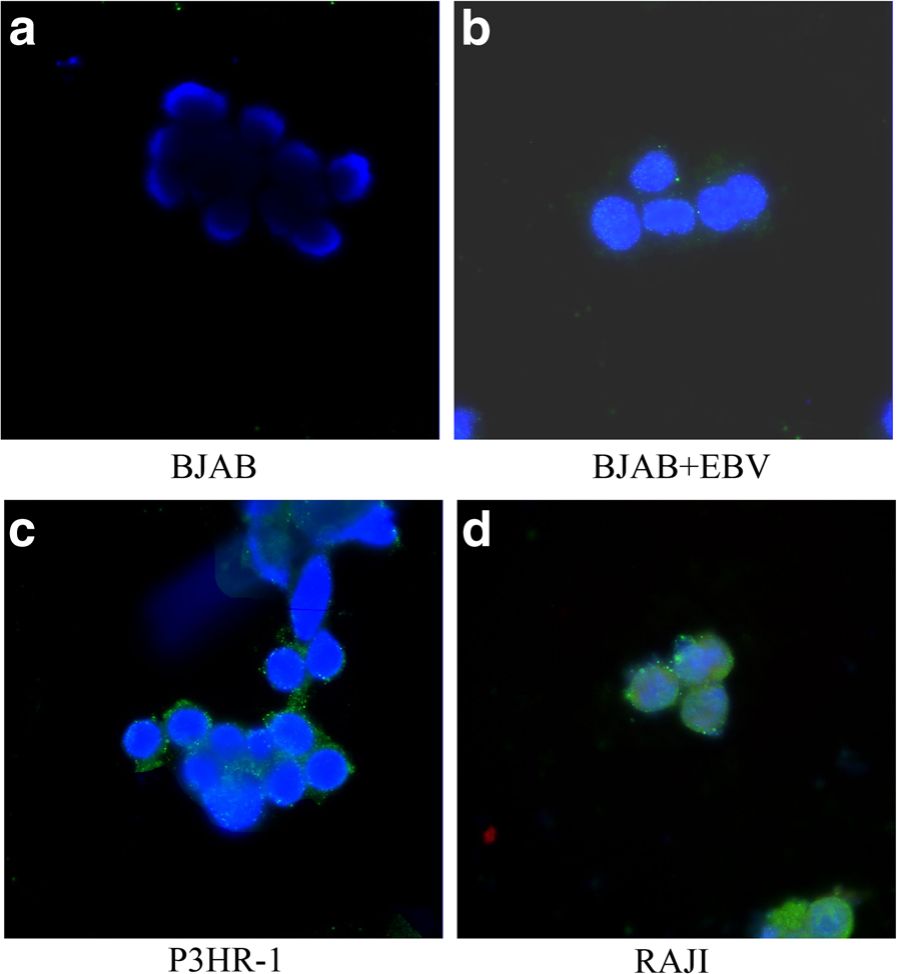
FISH assay for EBV in EBV positive and negative cell lines. Fluorescence in situ hybridization for detection of the EBV genome was performed in EBV-negative cell line BJAB (**a**), EBV-infected BJAB (**b**), and EBV positive cell lines P3HR-1 (**c**) and RAJI (**d**)

**Fig. 2:**
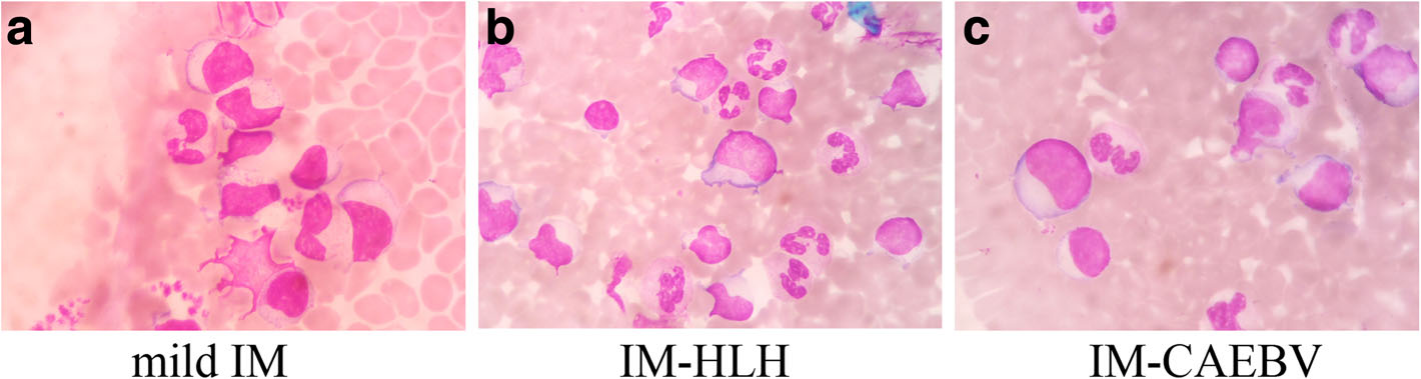
Atypical Lymphocytes in PBMCs of IM patients. **a**: Atypical lymphocyte in PBMC of mild IM patients; **b**: Atypical Lymphocyte in PBMC of IM-HLH; **c**: Atypical Lymphocyte in PBMC of IM-CAEBV (×1000, Wright–Giemsa)

1. **Specificity and sensitivity of FISH probe for EBV infection**
  The specificity and sensitivity of the FISH probe for EBV were determined in the EBV-negative cell line BJAB and EBV-positive cell lines P3HR-1, RAJI and EBV-infected BJAB. As shown in Fig. 1, in situ hybridization generated fluorescent spots in P3HR-1, RAJI and EBV-infected BJAB cells. However, in the EBV-negative BJAB cell line, no fluorescent particles were detected by FISH (Fig. 1). This suggests that hybridization with the 3267-bp EBV sequence probe was specific and sensitive for viral DNA.
2. **Molecular tests are superior to serological tests for monitoring EBV infection in IM**
  Atypical lymphocytes were found in 5–41% (median positivity rate of 17.8%) of PBMCs from mild IM patients, whereas 8–51% (median positivity rate of 21.6%) of PBMCs from IM-HLH and IM-CAEBV patients harbouredatypical lymphocytes (Fig. 2). There was no significant difference in the percentage of patients with more than 10% atypical lymphocytes between in the mild IM patients and the IM-HLH and IM-CAEBV patients (83.9% vs 85.7%, *p* > 0.05) (Table 2).
  Detection of IgM against VCA was performed with a semi-quantitative recombinant immunofluorescent antibody (RIFA) test on IM patients; 71.2% of mild IM patients and 85.7% of IM-HLH and IM-CAEBV patients were positive for EBV-VCA-IgM. EBV-VCA-IgM was negative in all healthy control patients. However, neither IM-HLH nor IM-CAEBV patients d had higher antibody titers against EBV-VCA than mild IM patients. In all, 67.1% of mild IM patients were heterophile antibody-positive, whereas 71.4% of IM-HLH and IM-CAEBV patients tested positive. Serum EBV DNA was defined using real-time PCR. EBV DNA was detected in 34 of 38 enrolled patients (89.5%), ranging from <500 to 8.26 × 10^7^ copies/ml. Four of the 38 clinically diagnosed IM patients were EBV DNA-negative according to plasma real-time PCR. Plasma samples obtained from the 20 control individuals were all EBV DNA negative. In situ hybridization for EBV DNA showed fluorescent spots in PBMCs obtained from 37 of 38 IM patients (positivity rate of 97.4%). According to the clinical diagnosis, the sensitivity values for atypical lymphocytes, the HA test, EBV-VCA-IgM, EBV DNA PCR and FISH were 84.2%, 68.4%, 76.3%, 89.5% and 97.4%, respectively. The sensitivity of the FISH test was significantly higher than that of atypical lymphocytes, the heterophile antibody test or EBV VCA-IgM (Table 2).
3. **FISH is superior to plasma EBV-DNA real-time PCR**
  The real-time PCR assay on the plasma of IM patients revealed that mild IM patients have fewer EBV genome copies than IM-HLH and IM-CAEBV patients (Table 3). Thirty-one of mild IM patients showed viral loads ranging from <500–9.37 × 10^5^ copies/ml, while 7 of IM-HLH and IM-CAEBV patients demonstrated viral loads ranging from 3.75 × 10^3^–8.26 × 10^7^ copies/ml. There was a significant difference between the mild IM patients and the IM-HLH and IM-CAEBV patients. However, plasma levels of EBV DNA cannot be directly linked to the severity of IM.

**Table 2:** The comparison of the sensitivity of serology tests and molecular tests

	Atypical Lymphocytes (>10%)	HA test	EBV-IgM	EBV PCR	FISH
mild IM(*n* = 31)	26/31(83.9%)	21/31(67.7%)	23/31(71.2%)	27/31(87.1%)	30/31(96.8%)
IM-HLH or IM-CAEBV(*n* = 7)	6/7 (85.7%)	5/7(71.4%)	6/7(85.7%)	7/7(100%)	7/7(100%)
Total(%)	32/38(84.2%)*	26/38(68.4%)^**^	29/38(76.3%)^*^	34/38(89.5%)	37/38(97.4%)

*Compare to FISH, *P* < 0.05; **Compare to FISH, *P* < 0.01

**Table 3:** Clinical features and laboratory results of the 38 IM patients

Num	Clinical type	HA test	EBV-VCA IgM	RT-PCR (copies/ml)	FISH positivity (per 100 cells)	FISH fluorescent	
						particles	outcome
1	mild	-	1:10	3.65×10^3^	61.5%	8.52/+	alive
2	mild	+	-	7.05×10^3^	3.0%	0.62/-	alive
3	mild	+	1:20	6.26×10^5^	66.0%	11.42/+	alive
4	mild	-	1:160	6.21×10^3^	54.5%	13.06/+	alive
5	mild	+	1:320	4.75×10^5^	56.5%	13.78/+	alive
6	mild	-	-	2.02×10^4^	62.0%	29.91/+	alive
7	mild	+	-	4.35×10^4^	42.5%	7.78/+	alive
8	mild	+	1:640	9.05×10^4^	65.0%	10.75/+	alive
9	mild	-	1:160	7.32×10^5^	57.5%	11.35/+	alive
10	mild	+	1:160	3.45×10^3^	72.5%	14.52/+	alive
11	mild	+	1:80	5.09×10^5^	63.0%	7.89/+	alive
12	mild	-	1:160	7.32×10^3^	56.5%	9.24/+	alive
13	mild	+	-	3.26×10^3^	70.5%	6.28/+	alive
14	mild	-	1:80	8.25×10^5^	50.5%	10.79/+	alive
15	mild	+	-	6.32×10^3^	63.0%	11.24/+	alive
16	mild	-	-	9.37×10^5^	41.5%	13.52/+	alive
17	mild	+	1:160	8.02×10^5^	67.5%	14.36/+	alive
18	mild	+	1:80	7.22×10^3^	70.5%	9.01/+	alive
19	mild	+	-	6.91×10^4^	52.0%	19.43/+	alive
20	mild	-	1:20	6.44×10^3^	45.5%	11.21/+	alive
21	mild	+	1:80	4.28×10^5^	35.5%	12.36/+	alive
22	mild	+	1:20	1.75×10^4^	45.5%	13.42/+	alive
23	mild	-	-	3.92×10^5^	72.5%	14.53/+	alive
24	mild	+	1:320	1.07×10^4^	65.5%	14.76/+	alive
25	mild	-	1:20	<500	42.0%	13.23/+	alive
26	mild	+	1:20	<500	53.0%	9.17/+	alive
27	mild	+	1:80	3.05×10^3^	67.0%	8.79/+	alive
28	mild	+	-	3.59×10^5^	71.0%	6.42/+	alive
29	mild	-	1:40	<500	39.0%	5.75 /+	alive
30	mild	-	1:20	<500	25.0%	12.31/+	alive
31	mild	-	1:320	2.63×10^4^	51.5%	14.53/+	alive
32	severe/EBV-HLH	+	1:160	3.75×10^3^	97.5%	32.82/+	alive
33	severe/EBV-HLH	-	1:320	4.5×10^7^	100%	36.66/+	deceased
34	severe/EBV-HLH	+	1:640	3.05×10^4^	89.5%	39.08/+	deceased
35	severe/EBV-HLH	+	-	5.05×10^4^	91.0%	51.03/+	alive
36	severe/CAEBV	+	1:40	8.92×10^6^	83.5%	53.44/+	alive
37	severe/CAEBV	-	1:320	8.26×10^7^	100%	55.34/+	deceased
38	severe/CAEBV	+	1:80	4.75×10^5^	87.5%	48.28/+	alive

**Fig. 3:**
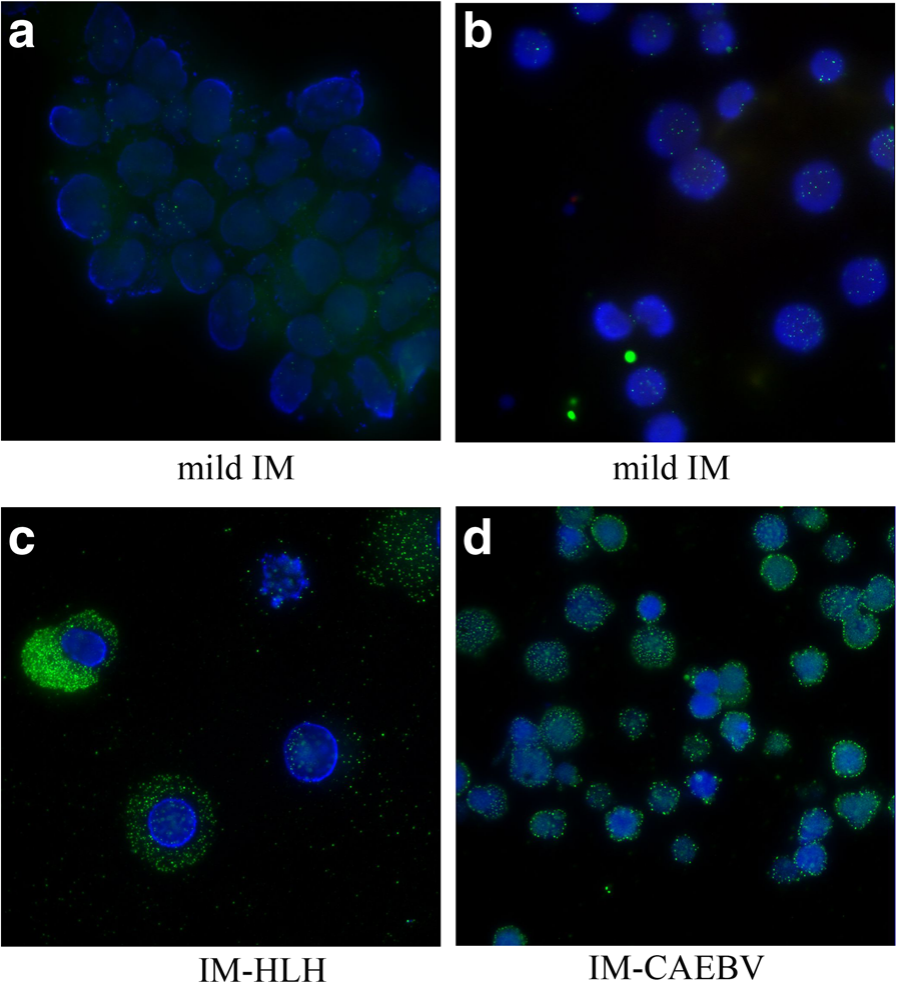
FISH assay for EBV in IM patients. Fluorescence in situ hybridization for detection of the EBV genome was performed in PBMC of IM patients. **a**, **b** mild IM patients; **c**, **d** IM-HLH or IM-CAEBV

The FISH assay was performed with PBMCs from IM patients. The genome copy number was much lower in the PBMCs from mild IM patients than the IM-HLH and IM-CAEBV patients. Compared to the cells in mild IM patients with 10 to 20 EBV genomes per cell (average fluorescent particles = 11.32), cells from IM-HLH and IM-CAEBV patients revealed 35 to 50 EBV genomes per cell (average fluorescent particles = 45.24), suggesting that the viral load increased with IM severity (Table 3 and Fig. 3, *P* < 0.05). The cut-off value of our EBV FISH was 4.02. EBV fluorescent particles were detected by FISH in 37 out of 38 IM patients. More than 90% (ranging from 83.5% to 100%) of PBMCs which may include T cells, B cells or NK cells, in IM-HLH and IM-CAEBV patients, were EBV viral DNA-positive according to the FISH test, whereas many fewer PBMCs were EBV viral DNA-positive in mild IM patients (3%–72.5%). This indicates that the method is good for the identification of IM with severe complications.

## Discussion

Some lymphoproliferative diseases (PTLD) are closely linked to EBV infection. However, infection of EBV is never associated with any symptoms in the majority of individuals. The association between the EBV infection status and lymphoproliferative disorders remains unknown. Stochastic loss of viral nucleic acids can be observed in EBV-related disease, suggesting the hit-and-run effects of EBV. EBV-driven malignant disease is closely linked to the viral latent state. IM is a mild lymphoproliferative disorder that is in most cases caused by EBV. Every organ can be involved in IM, andlife-threatening complications can occur. Diagnosis of IM is based on clinical, haematological findings and confirmed with a positive test for heterophile antibodies [13]. Serological tests are always used to analyze EBV-related antibodies. However, there was a greater variation in sensitivity and specificity. The seronegative window period varies among patients. The positivities obtained by the EBV-VCA-IgM and HA tests in this study are 76.3% and 68.4% for IM patients. Although some commercial serological tests for detection of EBV infection resulted in improvement in sensitivity and specificity, the titer against EBV-related antigens cannot be directly linked to the severity of IM and cannot differentiate between primary infection and reactivation [13]. Real-time PCR can provide a useful tool for the early diagnosis of IM in cases of inconclusive serological results [14]. At present, the best test for diagnosing and monitoring EBV infections is the blood viral load test, which is usually performed using a PCR platform [15]. The DNA of the virus detected by real-time PCR was detected in 89.5% of IM patients; 27 of 31 samples in the mild IM group and all samples in IM-HLH and IM-CAEBV group showed EBV DNA positivity by PCR. Detection of EBV by the FISH assay acquired 97.4% positivity, indicating that the molecular test more sensitively detects EBV infection, consistent with the conclusions of Horwitz [13] and Balfour HH’s [14]. In the present study, we found high numbers of genome copies detected by direct visualization of EBV genomes using in situ hybridization. In situ hybridization of the EBV DNA genome also provided a quantitative method to identify mild IM or IM-HLH and IM-CAEBV. The FISH assay also allows the in situ localization and visualization of spatial organisation of EBV as they infect cells in their natural habitat. Although the FISH assay detecting the EBV copies is expensive and time consuming, it still appears to be useful for diagnosis of IM and reactivation of EBV. The FISH methodologies can be improved by standardization and commercialization of EBV detection probe and in situ hybridization technique, allowing for the adaption to different laboratory’s operation. Although the small sample size puts a limitation on the study, there is significant difference in number of fluorescent spot between the mild IM patients and the IM-HLH and IM-CAEBV patients. Future research for gathering more date is needed.

EBV-infected B cells can go on to produce new virions, or the virus can enter a non-productive state known as latency. In the virus productive cycle, the EBV genome is amplified 100- to 1000-fold by the viral replication machinery. The link between EBV viral load and disease is yet to be determined. Monitoring of the EBV viral load in blood can be an effective method for distinguishing disease-associated EBV reactivation [16, 17]. Purposeful induction of the lytic form of EBV infection is now considered a strategy for the specific destruction of Epstein-Barr virus (EBV)-associated malignancies when the virus is latently infected. However, individuals who experience mononucleosis and high EBV viral load are at an increased risk of developing EBV-positive Hodgkin’s lymphoma [18]. The replication of EBV genomic DNA, which exists as a closed circular plasmid, is dependent on chromosomal initiation factors. The latent episome of EBV can also replicate for many generations without significant loss of copy number [19]. This indicates that the elevated viral DNA loads seen in these patients may be associated not only with lytic virus but also with latent virus. Plasma EBV DNA is considered an indicator for the staging and prognosis of nasopharyngeal carcinoma [20]. The EBV DNA load differs in Burkitt’s lymphoma patients and IM patients. In Burkitt’s lymphoma patients, the EBV DNA load was mainly situated in the cellular compartment, whereas in IM patients, the EBV burden in the circulation was almost exclusively restricted to the cellular blood compartment [21]. A more sensitive FISH than plasma EBV DNA PCR suggested a rapid disappearance of EBV DNA from plasma. The FISH assay detecting the EBV copies in unfractionated whole blood (which includes T cells, B cells and NK cells) is preferred and superior to plasma real-time PCR, as itreflects the absolute viral burden in the patient’s circulation. The FISH probe that is more than 3000 bp is preferablefor the detection of the absolute EB viral burden compared to probes that detect EBV-encoded RNAs (EBERs), which are considered a good test for localizing latent EBV in tissue samples.

## Conclusions

Taken together, Molecular tests, including FISH and EBV real-time PCR, are more sensitive than serological assays for the detection of EBV infection. FISH is a sensitive and specific tool for detecting EB viral burden. It can provide useful measurement for the early diagnosis of IM and has comprehensive clinical prospects and value.
